# Hyaluronan-Chondroitin Sulfate Anomalous Crosslinking Due to Temperature Changes

**DOI:** 10.3390/polym10050560

**Published:** 2018-05-22

**Authors:** Tomasz Andrysiak, Piotr Bełdowski, Jacek Siódmiak, Piotr Weber, Damian Ledziński

**Affiliations:** 1 Faculty of Telecommunications, Computer Science and Technology, UTP University of Science and Technology, Al. Prof. S. Kaliskiego 7, 85-796 Bydgoszcz, Poland; dledzinski@utp.edu.pl; 2 Institute of Mathematics and Physics, UTP University of Science and Technology, Al. Prof. S. Kaliskiego 7, 85-796 Bydgoszcz, Poland; piotr.beldowski@utp.edu.pl (P.B.); jacek.siodmiak@utp.edu.pl (J.S.); 3 Atomic and Optical Physics Division, Department of Atomic, Molecular and Optical Physics, Faculty of Applied Physics and Mathematics, Gdańsk University of Technology, G. Narutowicza 11/12, 80-233 Gdańsk, Poland; pweber@mif.pg.gda.pl

**Keywords:** hyaluronic acid, chondroitin sulfate, glycosaminoglycans, hydrogen bonds, physical crosslinking

## Abstract

Glycosaminoglycans are a wide class of biopolymers showing great lubricating properties due to their structure and high affinity to water. Two of them, hyaluronic acid and chondroitin sulfate, play an important role in articular cartilage lubrication. In this work, we present results of the all-atom molecular dynamics simulations of both molecules placed in water-based solution. To mimic changes of the physiological conditions, especially temperature, of the synovial fluid in joints under successive load (e.g., walking, jogging, jumping), simulations have been performed at different physiological temperatures in the range of 300 to 320 Kelvin (normal intra-articular temperature is 305 K). The stability of the biopolymeric network at equilibrium (isothermal and isobaric) conditions has been studied. To understand the process of physical crosslinking, the dynamics of intra- and intermolecular hydrogen bonds forming and breaking have been studied. The results show that following addition of chondroitin sulfate, hyaluronan creates more intermolecular hydrogen bonds than when in homogeneous solution. The presence of chondroitin in a hyaluronan network is beneficial as it may increase its stability. Presented data show hyaluronic acid and chondroitin sulfate as viscosity modifiers related to their crosslinking properties in different physicochemical conditions.

## 1. Introduction

**Figure 1:**
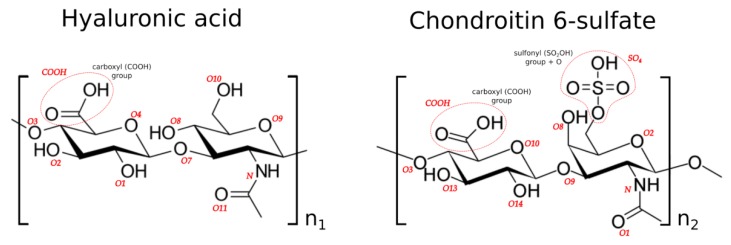
Chemical structures of the units in hyaluronic acid and chondroitin 6-sulfate chains. The red labels indicate the atoms and functional groups involved in the formation of hydrogen bonds.

Glycosaminoglycans are a group of long, unbranched polysaccharides built of repeating disaccharide units. Due to high polarity and water affinity, they can be found in many systems of human bodies. They occur on the surface of cells and in the extracellular matrix of animal organisms, such as skin, cartilage, lungs and so on. [1,2]. They are of particular importance as lubricants and as shock absorbers, which is one of the reasons for the remarkable functionality of the articular cartilage (AC) system [3]. Two major components of synovial fluid (SF) are hyaluronic acid (HA) and chondroitin sulfate (CS), presented in Figure 1.

HA is a naturally occurring biodegradable polymer with a variety of applications in medicine, including scaffolding for tissue engineering, dermatological fillers and viscosupplementation for osteoarthritis treatment. HA is available in most connective tissues in body fluids, such as synovial fluid and the vitreous humor of the eye [4].

CS is used for osteoarthritis, often in combination with other ingredients, including manganese ascorbate, glucosamine sulfate, glucosamine hydrochloride, or *N*-acetyl glucosamine. CS is also taken orally for HIV/AIDS, heart disease, heart attack, weak bones (osteoporosis), joint pain caused by drugs used to treat breast cancer, acid reflux, high cholesterol, muscle soreness after exercise, a bladder condition called interstitial cystitis, the bone disease called Kashin–Beck disease, and itchy and scaly skin (psoriasis). CS is also used in a complex with iron for treating iron-deficiency anemia [5].

HA is built of d-glucuronic acid and *N*-acetyl-d-glucosamine, linked via alternating β-(1→4) and β-(1→3) glycosidic bonds. CS is composed of a chain of alternating sugars: *N*-acetylgalactosamine and glucuronic acid. Both substances are used in the treatment of osteoarthritis [1]. This is due to the fact that long fractions of both glycosaminoglycans tend to crosslink [6]. Experiments show that the extracellular matrices they create have viscoelastic properties suitable for joints, especially those exposed to high loads [1].

Both molecules have been used for osteoarthritis (OA) treatment as viscosity modifiers. The efficiency of HA- and CS-based therapies is a controversial matter, as most recent studies show that there is little or no significant effect on improving AC functionality. There is, however, evidence that both molecules, when combined, can significantly improve the lubricating properties of SF. As SF composition changes with progression of OA, the concentration of both molecules decreases [7]. Moreover, HA shows significant changes of its polydispersity towards shorter chains [1,7,8]. Normal synovial fluid HA’s molecular weight ranges from ~4 kDa to ~8 MDa, wherein higher-molecular-weight species, which are selectively lost commensurate with osteoarthritis, are associated with lubricating properties. The effect is a reduced degree of physical crosslinking, which effects deterioration of viscoelastic properties of SF. There are several physiological changes occurring during AC functioning [9]. Namely, pH can vary between 6.9 to 8.1, and temperature of SF changes by 10 to 15 Celsius degrees during activity [10,11]. Also, ionic composition of fluid can be different at every stage of functioning [7,12,13]. The synergistic effect of HA–CS interactions can be one of the factors contributing to the decreasing friction coefficient of AC under mixed-mode lubrication conditions [12,14,15,16,17].

This work presents molecular dynamics study of HA–CS interactions as dependent on temperature. We look at hydrogen bonds as they play a key role in sustaining mechanical properties of the polymer network. A hydrogen bond map has been analyzed to identify atoms and/or functional groups which play the major role in extracellular matrix formation. As a result, our study suggests the molecular details of polymer network creation and maintenance as an explanation for the changing physiological condition.

## 2. Materials and Methods

### 2.1. Materials

The Simplified Molecular-Input Line-Entry System (SMILES) codes [18] of HA and CS structures were downloaded from the Open Chemistry Database PubChem (Bethesda, MD, USA) [19]. The long HA and CS chains were simulated using the YASARA Structure Software (Vienna, Austria) [20]—a molecular-graphics, -modeling and -simulation program powered by the PVL (Portable Vector Language). The effective molecular mass of HA was 160 kDa. In order to implement a water-based solvent, a four-site model (TIP3P) of water was used [21]. All atom simulations were performed under the following conditions: temperature: 300, 310 and 320 K, pH = 7.0 and in 0.9% NaCl aqueous solution. The time step has been set to 2 fs. Berendsen barostat and thermostat with a relaxation time of 1 fs were used to maintain constant pressure and temperature. HA forms a mesh-like network in synovial fluid solution and is not uniformly distributed over the entire volume. This is why regions with significantly higher than average (physiological) concentration are distinguished. The concentration of HA chosen was *C_HA_* = 10^−6^ M—much higher than the average in SF, since our focus was on the dense network region of SF, rather than the overall concentration. The concentration of CS was set at *C_CS_* = 4 × 10^−7^ M.

### 2.2. Methods

#### 2.2.1. Molecular Dynamics Force Field

All atom molecular dynamic simulations were performed using AMBER03 force field [22] to evaluate interactions between hyaluronan and chondroitin sulfate molecules. The AMBER03 potential function describing interactions among particles takes into account electrostatic, van der Waals, bond, bond angle, and dihedral terms:

(1) $$\begin{array}{l} E_{total}=\underset{bonds}{\sum }k_{b}{\left(R-R_{eq}\right)}^{2}+\underset{angle}{\sum }k_{\theta }{\left(\theta -\theta_{eq}\right)}^{2}+\underset{dihedrals}{\sum }\frac{V_{n}}{2}\left[1+\cos\left(n\varphi -\gamma \right)\right]\\ \;+\underset{i<j}{\sum }\left[\frac{A_{ij}}{R_{ij}^{12}}-\frac{B_{ij}}{R_{ij}^{6}}+\frac{q_{i}q_{j}}{\varepsilon R_{ij}}\right] \end{array}$$

where *k_b_* and *k_θ_* are the force constants for the bond and bond angles, respectively; *R* and *θ* are bond length and bond angle, respectively; *R_eq_* and *θ_eq_* are the equilibrium bond length and bond angle, respectively; *ϕ* is the dihedral angle and *V_n_* is the corresponding force constant; and phase angle *γ* takes values of either 0° or 180°. The nonbonded part of the potential is represented by van der Waals (*A_ij_*) and London dispersion terms (*B_ij_*), and interactions between partial atomic charges (*q_i_* and *q_j_*). *ε* is the dielectric constant that takes into account the effect of the medium that is not explicitly represented and usually equals 1.0 in a typical solvated environment, where solvent is represented explicitly. The nonbonded terms are calculated for all atom pairs that are either separated by more than three bonds, or are not bonded. Interactions between atoms separated by three bonds account for the one to four interactions in which the electrostatic and van der Waals parts are reduced by 20–50%, depending on the specific implementation of the force field. In this version, the one to four electrostatic interactions are divided by a factor of 1.20 and the Lennard–Jones terms are divided by 2.0 [22].

**Figure 2:**
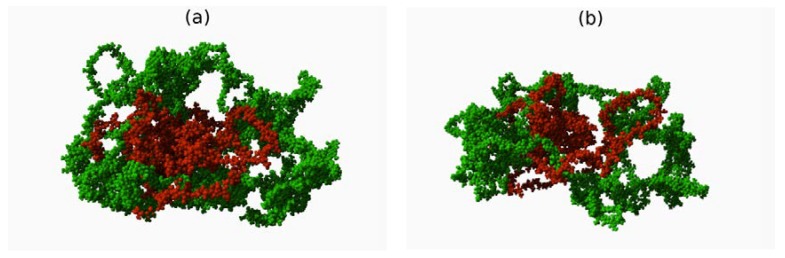
Initial (**a**) and final (**b**) structures of HA–CS complex. HA is colored green and CS is colored red. The addition of CS to the HA network causes the network to distend. The distended HA is able to create new intermolecular bonds (at the expense of intramolecular ones) which increase the stability of the network. Solvent molecules are not displayed for better visualization of HA and CS configuration.

The molecular initial (a) and final (after 10 ns molecular dynamics simulation) (b) structures of hyaluronan-chondroitin complexes are presented in Figure 2. As one can see, the system slightly expanded, yet it preserved its initial structure.

#### 2.2.2. Hydrogen Bond Identification and Strength

**Figure 3:**
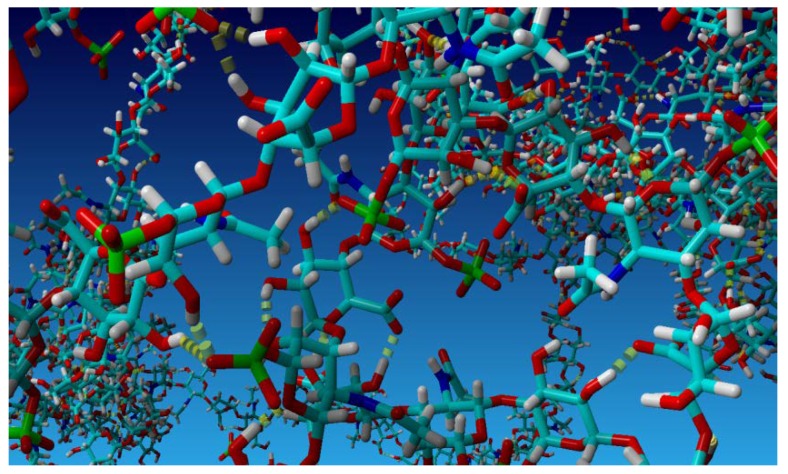
A fragment of the HA–CS network with H-bond visualized with lime dotted bars. Light blue atoms represent carbon, dark blue—nitrogen, red—oxygen, yellow—sulfur and white—hydrogen.

Hydrogen bonds are formed between two oxygen atoms, see Figure 3, if: (i) the distance between the hydrogen and adjacent oxygen atoms is smaller than 2.6 Å; and (ii) the distance between two neighboring oxygen atoms is less than 2.8 Å. Hydrogen bond energy, as defined by Equation (2), is greater than 6.25 kJ/mol (or 1.5 kcal/mol), which is 25% of the optimum value 25 kJ/mol. Thus, only strong and weak (up to 2.6 Å) are considered in the analysis. Equation (2) yields the bond energy in kJ/mol as a function of the hydrogen-acceptor distance and two scaling factors [20]:

(2) $$E_{HB}=25\cdot \frac{2.6-max\left(Dist_{H-A},2.1\right)}{0.5}\cdot Scale_{D-A-H}\cdot Scale_{D-A-X}$$

where the first scaling factor depends on the angle formed by donor–hydrogen-acceptor, and the second scaling factor is derived from the angle formed by hydrogen–acceptor–X, where the latter X is the atom covalently bound to the acceptor. Both scaling factors vary from 0 to 1.

### 2.3. Small-World Network Analysis

Molecular dynamics gives us a very detailed picture of phenomena [23]. Sometimes we expect only to extract the most important feature of these systems that follow from full atomic representation. This leads us to coarse-grain models [24,25,26]. The small-world networks theory can be considered as a tool that takes such a coarse-grain point of view on polymer properties and dynamics. There are examples of the effective use of this method in polymer science [27].

The small-world networks, in general, give an approach to the study of a topology of a complex dynamical system by representing it as an undirected graph. Such a graph consists of a set of N nodes and a set of edges that connect two elements of the sets of nodes. From the physical point of view, nodes can represent elements of the system, and interactions between constituent elements are represented by edges. The edges are assigned by numbers that can represent some relationships between parts of the system. We can look at this function like a kind of distance. Taking different definitions of “distances”, we can obtain various kinds of graphs. All definitions of distances in this work we take from the data collected from molecular dynamics simulations. For such a constructed graph, we obtain characteristic numbers within the theory of small-world networks. One such distance is taken as the number of edges along the shortest path connecting two nodes. For each graph there is a number called the characteristic path length of the network [28,29]. This number is defined as an average distance between two nodes calculated over all pairs of nodes.

In this work, we analyze intramolecular interactions inside the polymer molecules described above: HA and CS. We also analyze the HA–CS complex, but in this case, we consider the only interaction between chemical groups belonging to two different molecules: HA and CS. In the case of HA–CS, we do not analyze intramolecular interactions. In all cases, edges of the graph represent sets of a chemical group of one type in the molecule. For example, all carboxyl groups in one HA molecule compose a set. Moreover, each hydroxyl group presented in Figure 1 belongs to a different set of chemical group and so on. We can clearly define these sets because the considered molecules are polymers that are built of repeating units. Such a set we call a class, analogous to the class of abstraction known from mathematics. In the first case, each pair of nodes in the graph consists of two classes of chemical groups of the same molecule. In the case of complex HA–CS, each pair of nodes in the graph consists of one class of chemical group from the first molecule and the second class of chemical group from the second molecule.

We define three types of “intramolecular graphs” for molecular systems described above: HA and CS. We also define three “intermolecular graphs” for HA–CS complex. The first type of graph takes into account the length of the molecular distance between the selected chemical groups. In the case of HA and CS, molecule classes belong to one polymer molecule. In the second case, the considered classes of chemical groups belong to two different molecules and we do not consider intramolecular interactions. In all cases, the length of the distance between the two selected classes (two selected nodes in the graph) is a mean distance over all distances appearing in the calculation between two elements, each of which belongs to its own class. The second type of graph presents interaction energy between the chemical groups—an energy graph. The classes are the same as in the first type of graph, and are represented by the node in a graph. Each edge between the two classes gets a number that is calculated as a mean value of energy over all energies appearing in calculation between the two elements, each of which belongs to its own selected two classes. High absolute values of energy mean that interaction is strong, therefore, when we are looking for the shortest path in the graph we are looking for the path where the “chain of intramolecular/intermolecular interactions” has the largest value (as a sum of distances in energy domain). This type of graph distance we call “energy path”. The third type of graph also maintains the division into classes as in the first and second type of graph. Here we take into account the number of contacts between the chemical groups—number graphs. Each edge between the two nodes in this type of graph is assigned a number calculated as the average contact over all contacts appearing in the calculation between the two chemical groups, each of which belongs to its own selected two classes. This type of graph distance we called “number path”. Similar to the energy graph, we consider the most frequent contacts, so we multiply this value by (−1) and look for the minimum. This means that we are looking for the “chain of intramolecular/intermolecular interaction” when elements contact most frequently (as a sum of distances in contact domain). In the end, for three types of graphs, we calculate the characteristic path length of the network, according to the presented-above definition and meaning of path. In the algorithm we define the following attribution: If there is a zero node along the shortest connecting path then we put 1, if there is one node then we put 2, and so on. The results we present in Table 1 and Table 2. Energy analyzes are very time consuming and will be the subject of further research. This will allow a better understanding of intermolecular crosslinking processes.

## 3. Results

**Figure 4:**
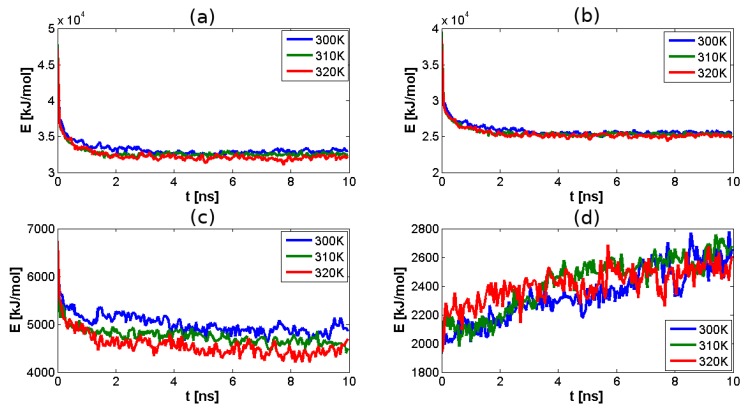
H-bond energy inside HA–CS network dependence on temperature: (**a**) total H-bond energy, for all studied temperatures the close-to-equilibrium state was reached after 2 ns; (**b**) H-bond energy between HA chains; (**c**) H-bond energy between CS chains; (**d**) H-bond energy between HA and CS chains. It can be seen that the formation of intermolecular bonds between HA and CS occurs at the expense of the amount of intramolecular CS bonds.

Data obtained from simulation show that there is high affinity between HA and CS molecules, as could be expected. The simulation time was 10 ns. As shown in Figure 4a, after 2 ns, the system reached close-to-equilibrium state. As presented in Figure 4a–d, total and intramolecular H-bond energies were obtained, however, intermolecular interactions did not reach a constant value. Comparing Figure 4c,d, it can be seen that the formation of intermolecular bonds between HA and CS occurs at the expense of the amount of intramolecular CS bonds.

**Figure 5:**
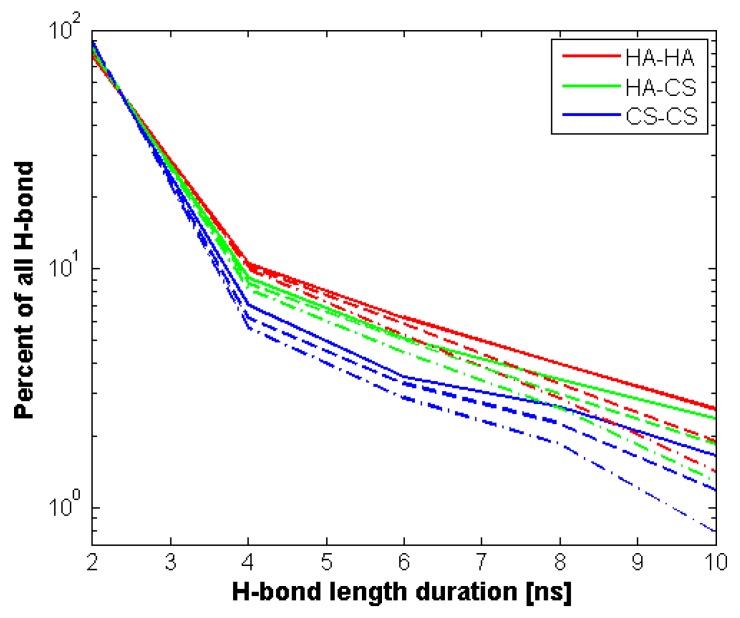
Inter- and intramolecular H-bond duration distribution. Line styles represent temperature: solid–300 K, dashed–310 K and dash dotted–320 K. It is seen that temperature plays a significant role in bond stability, lower temperatures leading toward more stable bonds.

**Figure 6:**
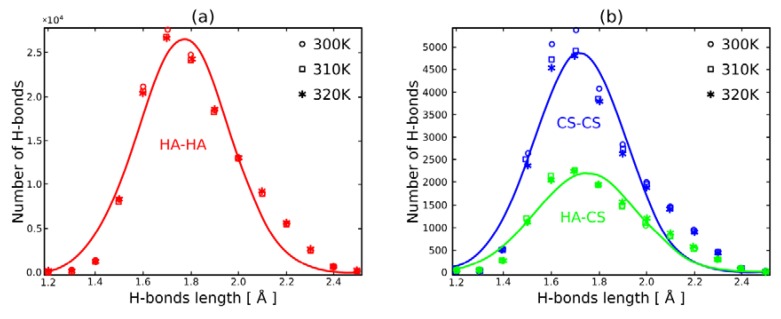
Hydrogen bonds length distribution: within HA ((**a**)-red); within CS ((**b**)-blue) and between HA and CS ((**b**)-green). The mean H-bonds lengths are: $d_{H_{HA-HA}}=1.77\pm 0.28$ with $R^{2}=0.95$, $d_{H_{CS-CS}}=1.72\pm 0.27$ with $R^{2}=0.93$ and $d_{H_{HA-CS}}=1.75\pm 0.30$ with $R^{2}=0.94$. Markers correspond to different temperatures: circles 300 K, squares 310 K and stars 320 K.

However, when we look into Figure 5 there is an apparent difference in terms of bond stability (understood as H–bond duration). Namely, 75% of hydrogen bonds formed by HA lasted at most 2 ns, while in the case of CS, this percentage is as much as 90%. Intermolecular bond duration is ~83% of this scale. Moreover, as one could predict, temperature plays a significant role in bond stability, lower temperatures leading toward more stable bonds. Figure 6 shows H–bond distribution length. It can be seen that both molecules show similar distribution, however due to its length, HA creates more bonds. In all cases, the temperature does not have a significant influence on the length of the bonds. The length distribution is the same as occurs in real systems and ranges from 1.5 to 2.5 Å.

**Figure 7:**
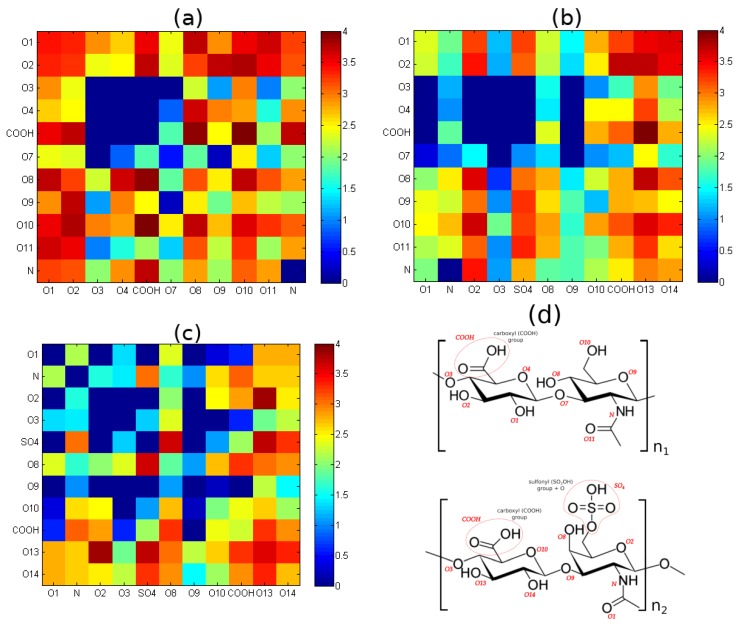
Hydrogen-bond energy map showing sites: (**a**) HA–HA interactions; (**b**) HA–CS; (**c**) CS–CS. Data present log(energy). Energy is presented in kJ/mol; (**d**) denotation of atoms and functional groups involved in the formation of hydrogen bonds in HA (**top**) and CS (**bottom**).

Finally, we looked into specific H-bonding sites between molecules, presented in Figure 7. As presented from left to right, HA intramolecular bonds are much less selective as only 10 out of all 66 possible do not occur during simulation. On the other hand, the CS–CS bond characteristic does not exhibit 17 of 66 possible pairs. Lastly, for HA–CS, 20 out of 121 are not present for the intermolecular case.

The small-world network analysis according to the algorithm presented in Section 2.3 has been performed. The results for length distance between chemical groups are shown in Table 1. This analysis has shown that the characteristic path length is smaller in the HA molecule (1.16–1.20) than in CS (1.42–1.45). Taking into account the algorithm, this means that statistically, the average number of nodes in the shortest intramolecular path is smaller for HA than for CS. The same trend is seen for number distance (values of characteristic path length, in the first column: 8.44–8.65, is smaller than in the third one: 8.87–8.96).

In the case of the complex HA–CS, we can see the dependence of all distances on temperature. In the length distance we see that for temperature 310 K, this value is greater than this value at the other two temperatures. In the case of number distance, we see that these values are the smallest at the temperature 310 K.

## 4. Discussion

The results presented here give more molecular details to mechanism of interaction, as they present the mechanism of H-bonds created between specific sites inside and between molecules of interest. HA is the most important molecule regarding its shock-absorbing properties; due to its length of over 3 MDa (for articular cartilage system), it can create a bigger network and thus provide an ideal platform for lubrication [30].

It has been shown that a distended matrix of HA–CS complexes, see Figure 2, can change viscoelastic properties of the lubricant that is synovial fluid [17]. This is because the HA matrix stretches as a result of interactions between HA and CS, and consequently, HA molecules can form more (at the expense of intramolecular ones) stabilizing intermolecular hydrogen bonds with other HA molecules. More intermolecular hydrogen bonds mean greater stability of the entire network. This in turn leads to the beneficial viscoelastic properties of the SF system [10]. As both molecules belong to the same group, they show high affinity towards each other. However, due to the presence of sulfate group in CS, they are not as efficient a lubricant as an HA molecule [30,31,32].

CS, due to its short length (less than 60 kDa), is rather an addition helping to sustain the network, as it can easily diffuse inside an HA network. Figure 5 shows H–bond duration of intra- and intermolecular bonds inside the network. HA shows longer-lasting networks (especially when comparing with pure HA solution [8]), and in total there are many more H-bonds than for CS and intermolecularly (see: Figure 6). This is rather due to the lower diffusivity of longer chains [33]. What can also be observed is that temperature has more impact on CS intramolecular bonds. This also may be the consequence of higher diffusivity of this (short) molecule than in the case of (long) HA [34].

However, the most interesting result is presented in Figure 7. It has been shown that although CS intramolecular bonds are more likely to occur, the intermolecular bonds with HA are less selective. This explains why the addition of CS to HA solution is beneficial for polymer network persistence. As it was suggested before, CS can diffuse more easily in the system and is more likely to form multiple bonds. There is another effect on both structures. Namely, H-bonds inside HA seem to contribute to chain stiffness, as most of them favor closest neighbors, such as bonds between the carboxyl group and atoms O8 and O10. On the other hand, CS seems to be much more flexible, as there are many fewer bonds of such type. Thus, its role would be to act as a temporary filler in the network. These intermolecular bonds are rather stable, thus, it can provide a certain level of efficiency. Taking into account small-world network analysis, defined at the beginning of our work, we can see that this method highlights the conclusions drawn from the maps.

## Figures and Tables

**Table 1:** Characteristic path length for chemical groups—length distance.

Temperature	Length Distance	Length Distance	Length Distance
	HA	HA–CS	CS
300 K	1.20	1.27	1.42
310 K	1.20	1.35	1.45
320 K	1.16	1.29	1.44

**Table 2:** Characteristic number path length for chemical groups—number distance for molecules HA, CS and complex HA–CS.

Temperature	Number Distance	Number Distance	Number Distance
	HA	HA–CS	CS
300 K	8.62	9.89	8.87
310 K	8.44	9.51	8.91
320 K	8.66	9.93	8.96

## Abbreviations

HA	Hyaluronic acid
CS	Chondroitin sulfate
SF	Synovial fluid
